# Initial D_2_ Dopamine Receptor Sensitivity Predicts Cocaine Sensitivity and Reward in Rats

**DOI:** 10.1371/journal.pone.0078258

**Published:** 2013-11-04

**Authors:** Kathryn E. Merritt, Ryan K. Bachtell

**Affiliations:** 1 Department of Psychology and Neuroscience, University of Colorado, Boulder, Colorado, United States of America; 2 Center for Neuroscience, University of Colorado, Boulder, Colorado, United States of America; 3 Institute for Behavioral Genetics, University of Colorado, Boulder, Colorado, United States of America; University of Chicago, United States of America

## Abstract

The activation of dopamine receptors within the mesolimbic dopamine system is known to be involved in the initiation and maintenance of cocaine use. Expression of the D_2_ dopamine receptor subtype has been implicated as both a predisposing factor and consequence of chronic cocaine use. It is unclear whether there is a predictive relationship between D_2_ dopamine receptor function and cocaine sensitivity that would enable cocaine abuse. Therefore, we exploited individual differences in behavioral responses to D_2_ dopamine receptor stimulation to test its relationship with cocaine-mediated behaviors. Outbred, male Sprague-Dawley rats were initially characterized by their locomotor responsiveness to the D_2_ dopamine receptor agonist, quinpirole, in a within-session ascending dose-response regimen (0, 0.1, 0.3 & 1.0 mg/kg, sc). Rats were classified as high or low quinpirole responders (HD_2_ and LD_2_, respectively) by a median split of their quinpirole-induced locomotor activity. Rats were subsequently tested for differences in the psychostimulant effects of cocaine by measuring changes in cocaine-induced locomotor activity (5 and 15 mg/kg, ip). Rats were also tested for differences in the development of conditioned place preference to a low dose of cocaine (7.5 mg/kg, ip) that does not reliably produce a cocaine conditioned place preference. Finally, rats were tested for acquisition of cocaine self-administration and maintenance responding on fixed ratio 1 and 5 schedules of reinforcement, respectively. Results demonstrate that HD_2_ rats have enhanced sensitivity to the locomotor stimulating properties of cocaine, display greater cocaine conditioned place preference, and self-administer more cocaine compared to LD_2_ animals. These findings suggest that individual differences in D_2_ dopamine receptor sensitivity may be predictive of cocaine sensitivity and reward.

## Introduction

Understanding why some individuals develop substance abuse or patterns of compulsive drug use while others do not is one of the most poorly understood aspects in the development of drug addiction. Epidemiological studies report that nearly 17% of people who use cocaine will become cocaine dependent within 10 years of initial cocaine use [1]. This suggests that some individuals are vulnerable, while others are resistant to developing drug dependence despite having a history of drug use. While there are many factors that may contribute to drug dependence (e.g. drug availability, social pressures, etc.), the discrepancy between vulnerable and resistant individuals may also be explained through individual differences in the functioning of the neurobiological systems underlying the responsiveness to drugs of abuse [2]. Understanding these differences may provide insight into one of the most sought after questions in the development of substance dependence.

The mesolimbic dopamine (DA) system consists of dopamine cells in the ventral tegmental area that project to medium spiny neurons in the nucleus accumbens among other limbic regions [3]. Cocaine rapidly elevates extracellular DA in the terminal regions of mesolimbic pathway by blocking the DA transporter, which contributes to cocaine reinforcement [4]. Activation of the mesolimbic pathway is widely known to be involved in the initiation and maintenance of cocaine use and use of other drugs of abuse [5]. Alterations within mesolimbic DA circuitry have been demonstrated as both a consequence of repeated psychostimulant use and as a predisposing factor. For example, chronic cocaine use is associated with decreased D_2_ DA receptor levels in the ventral striatum of cocaine abusers [6], suggesting that decreased D_2_ DA receptor expression is a consequence of chronic cocaine administration. There has been a long-standing debate about whether the decrease in D_2_ DA receptor expression observed in cocaine abusers is a result of chronic cocaine use or whether this alteration represents a pre-existing conditioning that may predispose an individual to develop cocaine dependence.

Recent work in humans and animals suggests that reduced D_2_ DA receptor expression may in fact be a vulnerability factor. Thus, non-addicted individuals with lower levels of D_2_ DA receptor report greater drug “liking” for the psychostimulant, methylphenidate [7]. Mutant mice lacking the D_2_ DA receptor self-administer more cocaine compared to wild-type animals [8], while over-expressing D_2_ DA receptors in the ventral striatum decrease cocaine self-administration [9]. Together these studies suggest that pre-existing alterations in D_2_ DA receptor expression may predict the reinforcing effects of cocaine, although there are still uncertainties concerning the specific role of D_2_ DA receptors as a vulnerability factor.

There is emerging interest in the dissociation between D_2_ DA receptor expression and D_2_ DA receptor function and sensitivity. While binge-like cocaine administration in rats recapitulates decreased D_2_ DA receptor expression, as observed in human cocaine abusers, there are somewhat paradoxical increases G protein activation in response to D_2_ DA receptor stimulation [10]. Likewise, cocaine self-administration increases the expression of high affinity D_2_ DA receptors [10], [11]. These changes suggest that while the expression of D_2_ DA receptors may decrease, the sensitivity of D_2_ DA receptors may increase following repeated cocaine. This notion is reflected in several behavioral paradigms where chronic cocaine produces cross-sensitization to the psychostimulant effects of D_2_ DA receptor agonists [12], [13], [14], [15], and stimulation of D_2_ DA receptors produces robust reinstatement to cocaine seeking in rodent self-administration models [16], [17], [18], [19], [20], [21]. It is unknown whether the pre-existing differences in the sensitivity of D_2_ DA receptors relate to the behavioral effects of cocaine.

In the present studies, we utilized a rodent model to identify how individual differences in the behavioral sensitivity of D_2_ DA receptors relate to cocaine-induced behaviors. Administration of the D_2_ DA receptor agonist, quinpirole, produces a high degree of variability in locomotor responses in drug naïve animals. Thus, we exploited these individual differences in the rat’s initial locomotor response to quinpirole as a model to test D_2_ DA receptor sensitivity as a vulnerability factor for subsequent cocaine-mediated behaviors. Those animals displaying robust increases in quinpirole-induced activity were characterized as having high D_2_ DA receptor sensitivity (HD_2_), while those rats having more modest activation were characterized as having low D_2_ DA receptor sensitivity (LD_2_)_._ Following this initial characterization, rats from each group were compared in cocaine-induced locomotion, cocaine-induced place preference, and cocaine self-administration.

## Materials and Methods

### Animals

Male Sprague–Dawley rats (Charles River, Portage, MI) weighing 275–325 g were individually housed upon arrival. Rats were given *ad libitum* food and water, except where indicated. All experiments were conducted during the light period of a (12∶12) light/dark cycle.

### Ethics Statement

These studies were carried out in accordance with the guidelines established by the Guide for the Care and Use of Laboratory Animals of the National Institutes of Health and were approved by the Institutional Animal Care and Use Committee at the University of Colorado at Boulder.

### Habituation to a Novel Environment

Locomotor activity was recorded in plexiglass chambers (San Diego Instruments, San Diego, CA, USA) measuring 16×16×15 in with 16 pairs of photobeams spaced 1 in apart across both horizontal planes. All locomotor tests were performed in unlit activity chambers during the light phase of the (12∶12) light/dark cycle. Animals were initially habituated to the novel locomotor testing chambers for 2 hrs prior to quinpirole-induced locomotor testing (see below).

### Characterization of the Quinpirole-induced Locomotor Behavior

The initial locomotor response to the D_2_ DA receptor agonist, quinpirole was used to classify animals into groups prior to any further behavioral testing. Tests began at least 7 days after the animals arrived from the vendor and were conducted in darkened locomotor chambers during the light period of a (12∶12) light/dark cycle. All animals were handled for approximately 5 min daily for 4 days prior to beginning these procedures to eliminate any potential interference. All animals were first habituated to the locomotor testing apparatus for 2 hrs the day prior to quinpirole testing (see above). Quinpirole-induced locomotion was assessed in a 5-hr within-session dose-response protocol as follows: 1-hr habituation followed by hourly ascending doses of the agonist (0, 0.1, 0.3 and 1.0 mg/kg, s.c.). A median split of total quinpirole-induced locomotor activity (calculated as Area Under the Curve, see below) was used to classify these rats as either high D_2_ responders (HD_2_) or low D_2_ responders (LD_2_). These procedures were conducted identically in several cohorts of animals (groups of rats arriving from the same vendor at identical age and weights) for each of the behavioral measures described (i.e. cocaine locomotion, place conditioning and self-administration). In each of the cohorts, the animal with the median score was tested, but eliminated from further data analyses. The distribution of scores within each cohort was qualitatively quite similar, but we did observe differences in the range and median scores for quinpirole-induced locomotor activity between cohorts of animals. Therefore, HD_2_ and LD_2_ classifications were made within each individual cohort.

### Cocaine-induced Locomotor Behavior

In one cohort of animals (N = 39), locomotor responses were measured using a 3-hr within-session cocaine dose-response protocol. These assessments were performed in darkened locomotor chambers during the light period of a (12∶12) light/dark cycle. Animals were tested 5–7 days following the initial characterization of their quinpirole sensitivity in the same activity chambers. On the test day, animals were habituated to the locomotor chamber for 1 hr and were then administered hourly ascending doses of cocaine (5 and 15 mg/kg, i.p.).

### Cocaine Place Conditioning

In another cohort of animals (N = 37), place conditioning was measured in an unbiased 3-chamber apparatus using an unbiased 3-phase procedure. Testing began 7 days following the initial characterization of quinpirole sensitivity. The two conditioning chambers (15 cm×25 cm×35 cm) were distinct in wall patterns (gray vs. vertical white and black stripes) and floor textures (grid vs. hole). The center compartment (15 cm×10 cm) had white walls and a plexiglass floor. Chambers are equipped with infrared photocells to detect animal position and movement in the apparatus. From 1000–1500 hrs on the day before conditioning (pre-conditioning), rats were allowed access to all three compartments for 20 min to test for initial bias. One animal was excluded from the experiment because it displayed an initial bias of 92% time in one compartment. Rats received three 30-min saline conditioning sessions and three 30-min cocaine (7.5 mg/kg, i.p.) conditioning sessions. Saline conditioning occurred between 0800–1100 hrs, while cocaine conditioning occurred between 1500–1700 hrs. The 7.5 mg/kg cocaine dose was chosen because preliminary studies in our lab demonstrate that it does not reliably produce a place preference in all rats. Therefore, this cocaine dose was ideal to identify potential differences in the development of a place preference between the two groups. The final test session (post-conditioning) was conducted between 1000 hrs and 1500 hrs and rats were again allowed free access to the three compartments and preference was determined as time spent in the drug compartment minus time spent in the saline compartment (conditioned place preference (CPP) score).

### Sucrose and Cocaine Self-administration

Another cohort of animals (N = 29) was tested for operant responding for sucrose pellets following the initial characterization of quinpirole sensitivity. Self-administration procedures were performed in operant conditioning chambers (Med-Associates, St Albans, VT) equipped with two response levers. Seven days following the initial quinpirole testing, these rats were food-restricted to prevent weight gain, and trained to lever-press for sucrose pellets on a fixed ratio 1 (FR1) reinforcement schedule until acquisition criteria had been achieved (50 sucrose pellets). The latency to reach this criterion was used as the dependent variable in these experiments. All rats reached criterion after approximately 8 days of training and were fed *ad libitum* thereafter.

Following the sucrose self-administration and at least one day of *ad libitum* feeding, animals were implanted with jugular catheters under halothane anesthesia (1–2.5%), as described elsewhere [22]. After 5–7 days of recovery from surgery, animals self-administered cocaine (0.5 mg/kg/100 µl, iv) under a FR1, timeout 20 s reinforcement schedule during 6 daily 2-h sessions. Animals were then transferred to a FR5, timeout 20 s schedule of reinforcement for an additional 5 daily 2-h sessions. Cocaine infusions were delivered over 5 s concurrent with the termination of the house light and illumination of a cue light above the drug-paired lever.

### Drugs

Quinpirole [(-)-Quinpirole hydrochloride] and cocaine hydrochloride were purchased from Sigma (St. Louis, MO). All drugs were dissolved in sterile-filtered physiological (0.9%) saline.

### Data Analysis

Cocaine-induced locomotor data (beam breaks) were analyzed by 2-factor mixed design ANOVA with quinpirole group (HD_2_ and LD_2_) and cocaine dose (5 & 15 mg/kg) as factors. Linear regressions were also performed on the locomotor data to identify the explanatory power of the quinpirole sensitivity in cocaine locomotion. Place conditioning data (CPP score = drug-paired minus saline-paired) was analyzed using a 2-factor mixed design ANOVA with quinpirole group (HD_2_ and LD_2_) and conditioning (Pre-conditioning and Post-conditioning) as factors. Cocaine self-administration data (cocaine infusions) were analyzed by both a 2-factor mixed design ANOVA with quinpirole group (HD_2_ and LD_2_) and days as factors, or an independent t-test between the quinpirole groups (HD_2_ and LD_2_) when cocaine infusions were collapsed across days. In all cases, significant main and interactive effects were followed by simple effects analyses and post hoc tests (Bonferroni’s test of significance). Statistical significance was preset at *p*<0.05.

## Results

### Characterization of High and Low Quinpirole Sensitivity Groups

**Figure 1:**
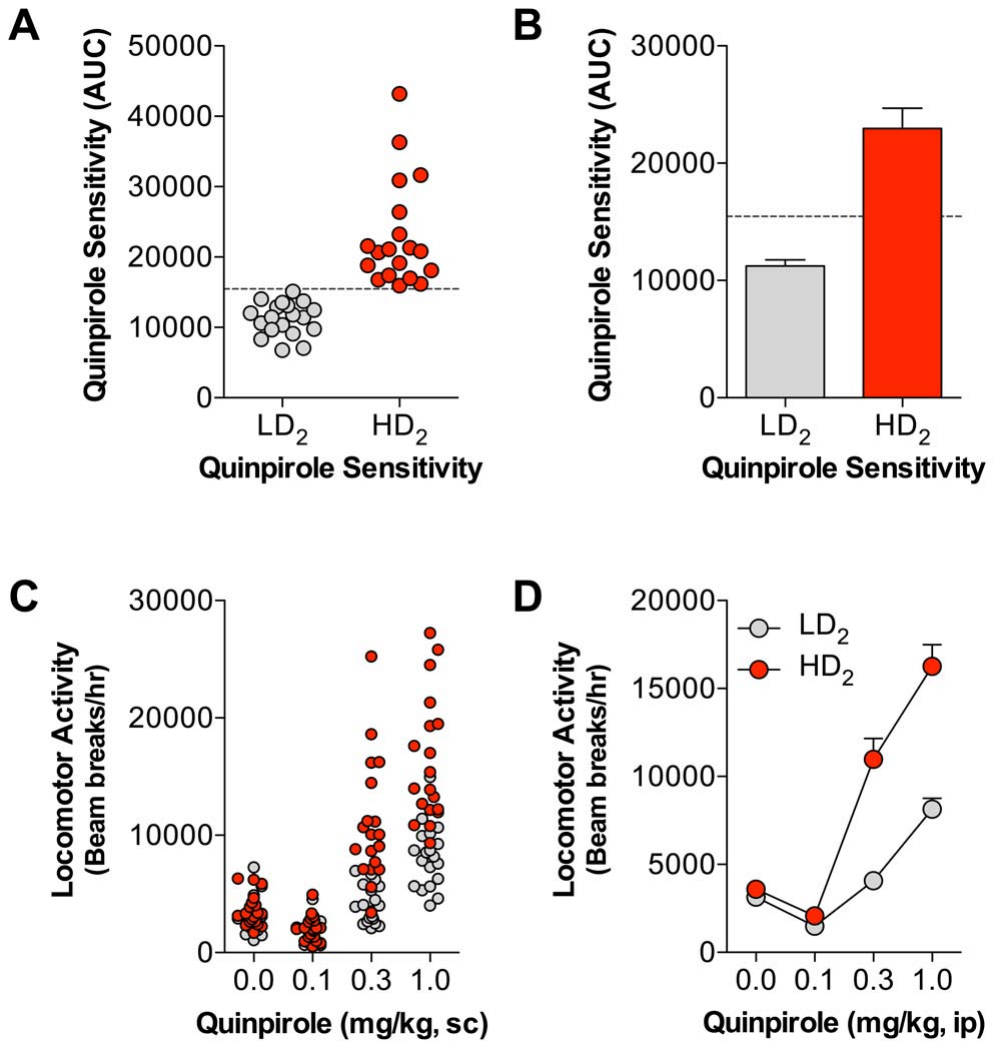
Distributions and averages of quinpirole-induced locomotor activity for LD_2_ and HD_2_ groups. (A) Group distributions of the calculated quinpirole area under the curve (AUC) scores used to classify rats into the LD_2_ and HD_2_ groups. The dotted line represents the median score (*M* = 15460). (B) Group averages (± sem) of the quinpirole AUC score used to generate the LD_2_ and HD_2_ groups. The dotted line represents the median score (*M* = 15460). (C) Distribution of locomotor activity scores (beam breaks/hr) during the ascending within-session quinpirole dose response testing within the LD_2_ (gray circles) and HD_2_ (red circles) groups. (D) Group averages (± sem) of the quinpirole dose response curve for the LD_2_ and HD_2_ groups.

There is a high degree of variation in responding across each quinpirole dose during the within-session dose response locomotor activity testing (Figure S1). Generally, the lowest dose of quinpirole (0.1 mg/kg, sc) suppresses locomotion compared to vehicle responding, while the higher doses (0.3 and 1.0 mg/kg, sc) activate locomotion. This is a prototypical quinpirole dose response, where low doses of quinpirole presumably stimulate D_2_ autoreceptors on dopamine terminals and higher quinpirole doses saturate D_2_ autoreceptors and stimulate postsynaptic D_2_ receptors [23], [24], [25]. In an attempt to capture the behavioral complexity of pre- and postsynaptic D_2_ receptor stimulation, we calculated the area under the curve (AUC) for each animal across all quinpirole doses (Figure S1). The quinpirole AUC score was then used to segregate each cohort into high quinpirole sensitivity (HD_2_) and low quinpirole sensitivity (LD_2_) groups based on a median split of the entire cohort. Figure 1A and 1B illustrate both the distribution of the quinpirole AUC scores and the group means following the median split into HD_2_ and LD_2_ groups. Figure 1C and 1D shows the distributions and group means of locomotion at each quinpirole dose. In developing the groups, the rat corresponding to the median score was eliminated from further analysis, but is shown on the graph to depict both the individual and mean range from the median score.

Given that the group assignments are primarily influenced by locomotor activation produced by quinpirole activation of postsynaptic D_2_ receptors, we also wanted to identify whether the groups differed in their responsiveness to the low, locomotor suppressing dose of quinpirole (0.1 mg/kg). To fully capture the magnitude of the suppressive effects of the low quinpirole dose, we calculated the suppressive effects of quinpirole as a percent of baseline (saline-induced activity; Figure S2). There were no differences in the quinpirole-induced locomotor suppression produced by 0.1 mg/kg quinpirole (t_36_ = 1.01, p = 0.3183), suggesting that the differential sensitivity to quinpirole between the HD_2_ and LD_2_ animals largely reflects the sensitivity of postsynaptic D_2_ DA receptors.

### High Quinpirole Sensitivity Predicts Increased Cocaine-induced Locomotion

**Figure 2:**
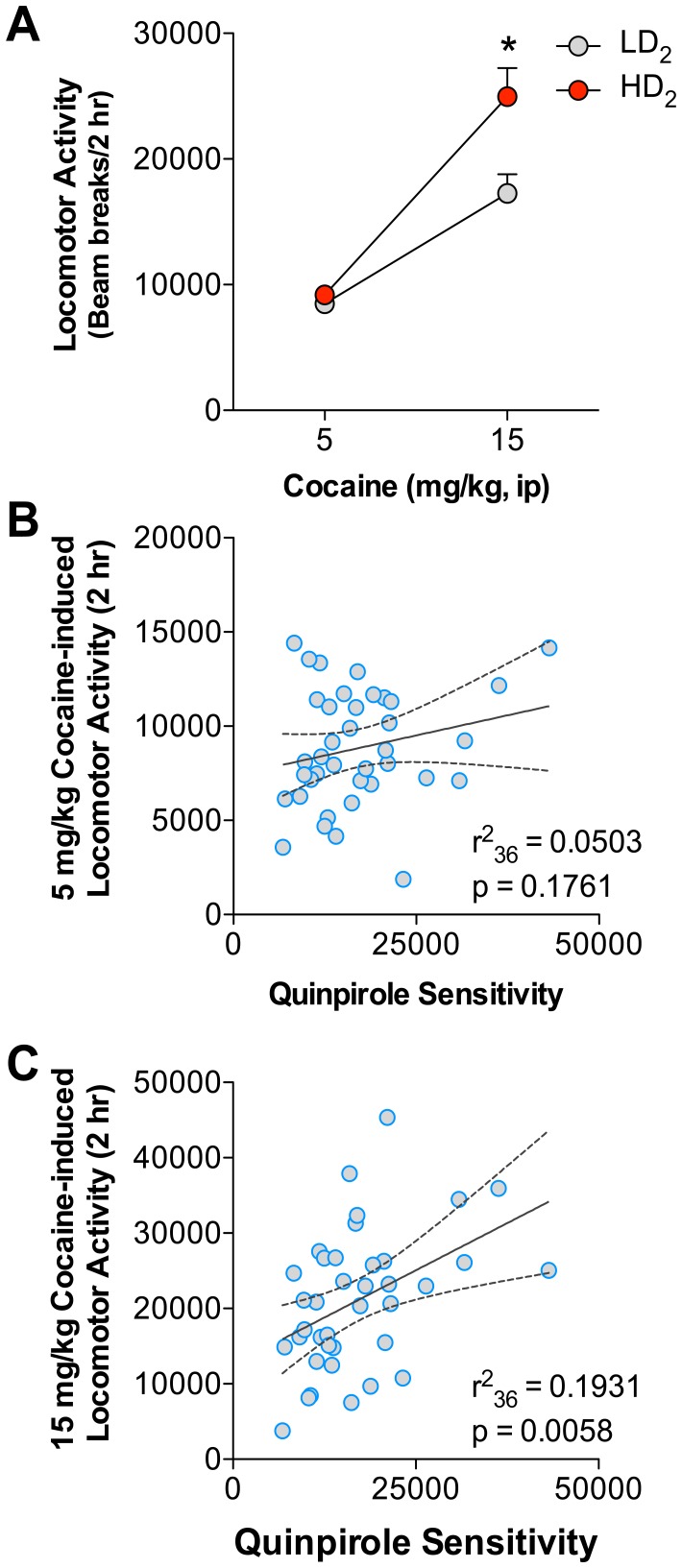
HD_2_ animals display greater sensitivity to cocaine-induced locomotor activity. (A) Rats were tested across two cocaine doses (5 and 15 mg/kg, ip) in a within-session procedure. HD_2_ animals displayed significantly greater cocaine-induced locomotor activity to 15 mg/kg cocaine, but not 5 mg/kg cocaine. *HD_2_ significant from LD_2_, p<0.05 (B and C) Analyses of the entire cohort were conducted to determine the relationship between quinpirole AUC scores and cocaine-induced locomotion. A non-significant positive relationship was identified for cocaine-induced activity at the low dose (B, 5 mg/kg cocaine) and a significant positive relationship was identified for cocaine-induced activity at the high dose (C, 15 mg/kg cocaine).

Utilizing the median split group assignments for quinpirole responding, we tested whether quinpirole sensitivity was related to the locomotor activating properties of cocaine. Figure 2 illustrates that HD_2_ animals had greater cocaine-induced locomotor activity following the 15 mg/kg cocaine dose, but not following the 5 mg/kg cocaine dose. A two-way mixed design ANOVA of these data reveal a significant interaction between cocaine dose and quinpirole group (F_1,36_ = 7.17, p = 0.0111), and main effects of cocaine (F_1,36_ = 88.43, p<0.0001) and group (F_1,36_ = 6.86, p = 0.0128). Figure 2 also displays the results of linear regressions performed at each cocaine dose across the entire population of animals. There was a significant relationship between quinpirole sensitivity and 15 mg/kg cocaine-induced locomotor activity (F_1,36_ = 8.62, p = 0.0058), but not 5 mg/kg cocaine-induced locomotor activity (F_1,36_ = 1.91, p = 0.1761). Thus, initial quinpirole sensitivity appears to predict cocaine-induced locomotion to a high, locomotor activating dose of cocaine.

**Figure 3:**
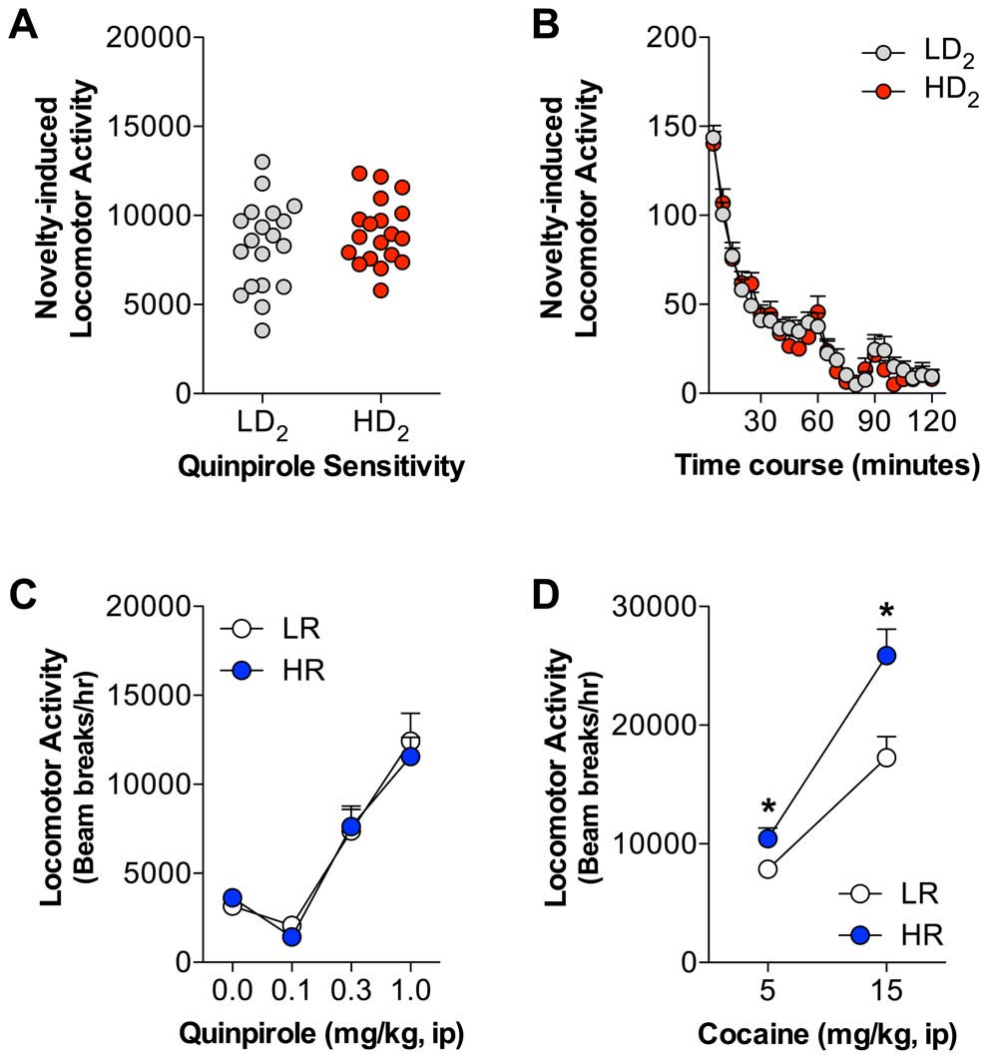
Quinpirole sensitivity is not associated with novelty-induced locomotor activity. Assessing novelty-induced locomotion during the habituation phase of testing revealed no significant differences between the LD_2_ and HD_2_ groups. (A) Distribution of novelty-induced locomotor activity scores over the 2-hr testing period. (B) Time course depicting novelty-induced locomotor activity between the LD_2_ and HD_2_ groups. Animals from this cohort were re-classified into a low responder group (LR) and high responder group (HR) based on their novelty-induced locomotor activity. (C) LR and HR rats did not predict differences in locomotor activity across the quinpirole dose response testing. (D) HR rats displayed significantly greater cocaine-induced locomotor activity across both cocaine doses. *HR significant from LR, p<0.05.

Previous work demonstrates that novelty-induced locomotion is predictive of future cocaine responding [26], [27]. Therefore, we wanted to assess if there were differences between LD_2_ and HD_2_ groups in novelty-induced locomotor activity. There was no difference between the HD_2_ and LD_2_ groups in novelty-induced locomotion across the entire session (Figure 3A: t_36_ = 0.44, p = 0.6601) or within the first 30–60 minutes (Figure 3B), when differences in novelty responsiveness are typically most robust. To identify whether novelty-induced locomotor activity was predictive of D_2_ DA receptor sensitivity, we re-characterized our rats as having either low or high novelty-induced locomotor activity. Thus, we created low responding rats (LR) and high responding rats (HR) based on a median split of their initial locomotor responsiveness to the locomotor testing apparatus during the habituation phase of testing. We then determined whether these groups differed in quinpirole-induced locomotor activity. As shown in Figure 3, LR and HR rats did not differ significantly at any of the quinpirole doses (Group: F_1,108_<1, NS; Quinpirole: F_3,108_ = 69.61, p<0.0001; Interaction: (F_3,108_<1, NS), although the groups did significantly differ in cocaine-induced locomotion (Group: F_1,36_ = 10.49, p = 0.0026; Cocaine: F_1,36_ = 84.86, p<0.0001; Interaction: (F_1,36_ = 5.02, p = 0.0313). Together, these data suggest that while novelty-induced locomotion is predictive of cocaine responsiveness, the mechanisms associated with this relationship may be distinct from those associated with D_2_ DA receptor sensitivity.

**Figure 4:**
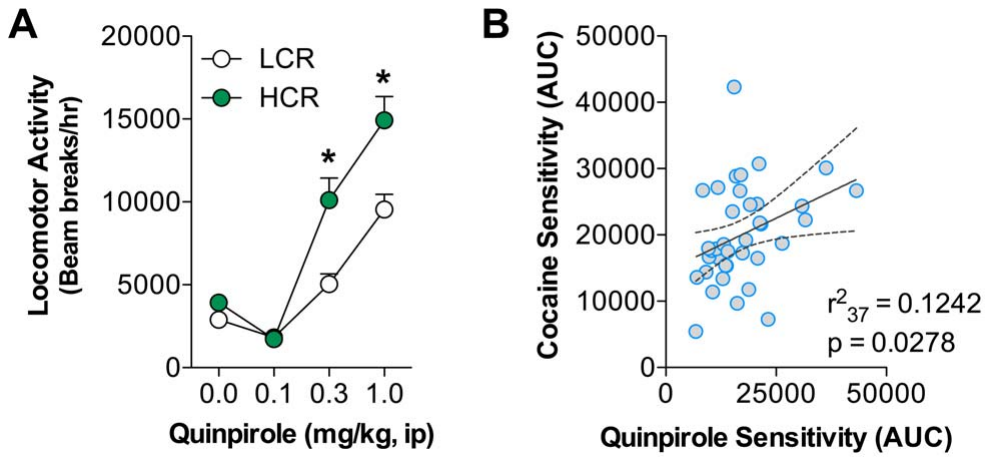
Initial cocaine sensitivity corresponds with differences in D_2_ DA receptor sensitivity. The area under the curve (AUC) was calculated for each rat’s cocaine-induced locomotor activity across both 5 and 15 mg/kg doses. Using this calculated score for initial cocaine-induced locomotor activity, rats were re-classified into a low cocaine responder group (LCR) and a high cocaine responder group (HCR). (A) HCR rats displayed significantly greater quinpirole-induced locomotor activity at the 0.3 and 1.0 mg/kg doses. *HCR significant from LCR, p<0.05. (B) An analysis of the entire cohort was conducted to determine the relationship between quinpirole AUC scores and cocaine AUC scores. A significant positive relationship was identified between initial quinpirole sensitivity and initial cocaine sensitivity.

Since individual differences in the initial locomotor response to cocaine have also been shown to correspond with alterations in the development of cocaine sensitization, cocaine reward and cocaine self-administration, we re-characterized our rats as having either low or high cocaine-induced locomotor activity [28], [29], [30], [31]. This re-characterization was based on calculating the AUC for cocaine-induced locomotion across both cocaine doses during the within-session cocaine dose response testing. Rats having AUC values below the median were placed in the low cocaine responder (LCR) group while those having AUC values above the median were placed in the high cocaine responder (HCR) group. We then determined whether initial cocaine-induced locomotion was predictive of quinpirole-induced activity. HCR rats had greater overall quinpirole-induced activity compared to LCR rats using the quinpirole AUC score (t_36_ = 3.585, p<0.0010, data not shown). Analysis of the activity across the quinpirole dose response testing suggests that these differences were primary observed at the locomotor activating quinpirole doses (Figure 4). Thus, analysis of the quinpirole dose response between the groups revealed a significant main effects of group (F_1,108_ = 14.05, p = 0.0006), quinpirole dose (F_3,108_ = 85.93, p<0.0001) and the interaction (F_3,108_ = 7.64, p = 0.0001). We also assessed the relationship between the overall cocaine sensitivity and quinpirole sensitivity using the AUC scores for each drug where there was a significant correlation between the two activity scores (Figure 4). Together these findings suggest that there is significant overlap between the initial cocaine sensitivity and initial quinpirole sensitivity.

### High Quinpirole Sensitivity Predicts Increased Cocaine Reward

**Figure 5:**
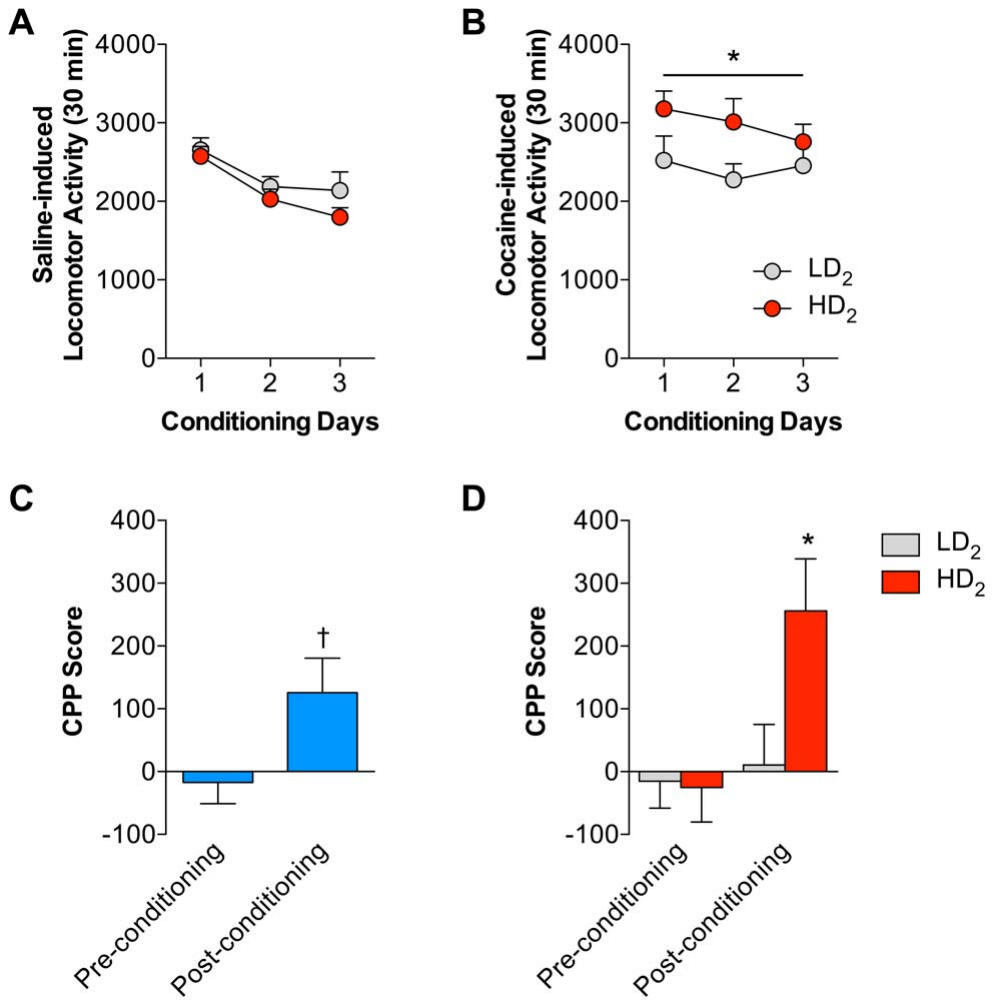
HD_2_ animals display greater sensitivity to the rewarding effects of cocaine. (A) There were no group differences in the saline-induced locomotor activity during the conditioning trials. (B) There was a significant group difference in the cocaine-induced activity during the conditioning trials where HD_2_ animals displayed significantly greater cocaine-induced locomotor activity across all session. *HD_2_ significant from LD_2_, p<0.05. (C) Analyses of all animals in the cohort demonstrated a significant, modest cocaine-induced place preference following conditioning. † Post-conditioning significant from pre-conditioning, t_36_ = 2.27, p = 0.0295. (D) Group analyses demonstrated that only animals in the HD_2_ group developed a significant preference for the cocaine-paired compartment compared to animals in the LD_2_ group that did not develop any significant conditioning to the cocaine-paired compartment. *HD_2_ significant from LD_2_, p<0.05.

In a separate cohort of animals, median split group assignments for quinpirole responding was created (data not shown) and place conditioning for cocaine (7.5 mg/kg) was tested. This dose was used in this test because it does not reliably produce robust place conditioning in all animals. Figure 5 illustrates both the saline- and cocaine-induced locomotion during the 30 min conditioning sessions. There was no significant group difference in saline-induced locomotion (F_1,66_ = 0.51, p = 0.4784). There was a significant decrease in saline-induced locomotion across each conditioning session (F_2,66_ = 10.91, p<0.0001) although there was no significant interaction between groups and sessions (F_2,66_ = 0.59, p = 0.5567). HD_2_ rats had significantly higher cocaine-induced locomotion during the conditioning sessions compared to LD_2_ rats (F_1,66_ = 4.29, p = 0.0462). There was no main effect of session (F_2,66_ = 0.77, p = 0.4595) and no significant interactive effects (F_2,66_ = 0.60, p = 0.5535), although qualitatively there appeared to be enhanced cocaine-induced locomotion during the first two conditioning sessions (Figure 5). Heightened cocaine-induced locomotion in HD_2_ animals during the conditioning sessions recapitulates our previous findings (Figure 2) and indicates that HD_2_ animals are more sensitive to the locomotor stimulating properties of cocaine and that may be predictive of cocaine reward. When the entire cohort was analyzed for the development of a conditioned place preference for cocaine, there was a significant increase in time spent in the cocaine-paired compartment post-conditioning (t_36_ = 2.27, p = 0.0295). When group was included in the analysis, there was a significant main effect of conditioning (F_1,34_ = 6.31, p = 0.0169), again suggesting that overall, animals developed a preference for the cocaine-paired compartment. There was no group effect (F_1,34_ = 3.27, p = 0.0793), but there was a significant interaction between conditioning and group (F_2,34_ = 4.36, p = 0.0443). Subsequent analyses revealed that HD_2_ animals displayed greater conditioned place preference to 7.5 mg/kg cocaine compared to LD_2_ animals on the post-conditioning test (t_34_ = 2.33, p = 0.0258), but did not differ on pre-conditioning test (t_34_ = 0.31, p = 0.7619). These findings suggest that initial quinpirole sensitivity is associated with heighted cocaine reward.

### High Quinpirole Sensitivity Predicts Increased Cocaine Self-administration

**Figure 6:**
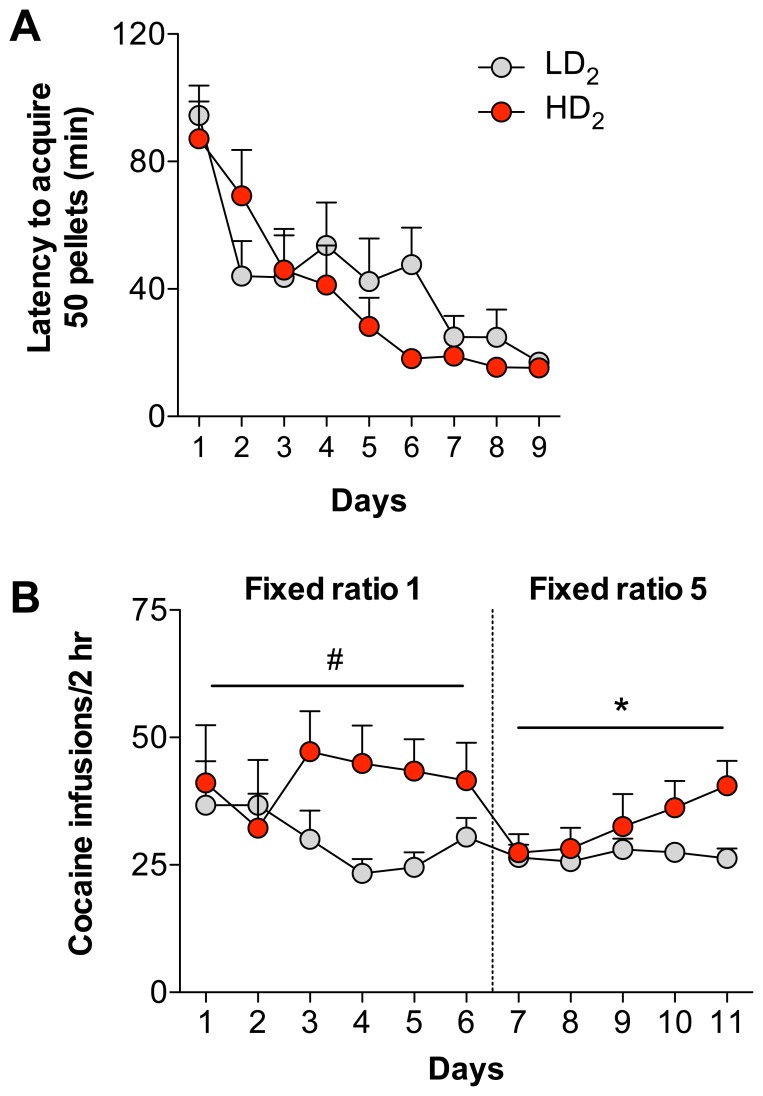
HD_2_ animals self-administer more cocaine than LD_2_ animals. (A) There were no group differences in the acquisition of an operant response to acquire sucrose pellets. (B) There were significant group differences in the number of cocaine infusions delivered on both a fixed ratio 1 and fixed ratio 5 schedule of reinforcement. ^#^significant trend between HD_2_ and LD_2_ groups, p = 0.08, *HD_2_ significant from LD_2_, p<0.05.

In a separate cohort of animals, median split group assignments for quinpirole responding was created and self-administration of either sucrose or cocaine was tested. Figure 6 illustrates that there was no group difference in the acquisition of sucrose self-administration (F_1,176_ = 0.39, p = 0.5406) and both groups acquired equivalently (Sessions: F_8,176_ = 18.00, p<0.0001; Group×Session Interaction: F_8,176_ = 1.81, p = 0.0775), suggesting that these groups do not differ in reinforced learning of an operant response. These same animals were then implanted with a chronic indwelling catheter and allowed to self-administer cocaine. Animals initially acquired cocaine self-administration on an FR 1 schedule. There was a trend for HD_2_ to self-administer more cocaine than LD_2_ animals on an FR 1 schedule analyzed across all sessions (F_1,95_ = 3.31, p = 0.0846). When sessions were averaged across all FR 1 sessions, HD_2_ animals self-administered significantly more cocaine than LD_2_ animals (t_19_ = 2.63, p = 0.0164, data not shown). When the schedule was advanced to an FR 5 schedule of reinforcement HD_2_ animals self-administered more cocaine across sessions as revealed by a significant interaction (F_4,76_ = 3.465, p = 0.0118), although this effect was not observed when averaged across all FR 5 sessions (t_19_ = 1.51, p = 0.1484, data not shown). Thus, enhanced initial quinpirole sensitivity is associated with increased cocaine intake.

### Cocaine Increases Quinpirole Sensitivity in both HD_2_ and LD_2_ Animals

**Figure 7:**
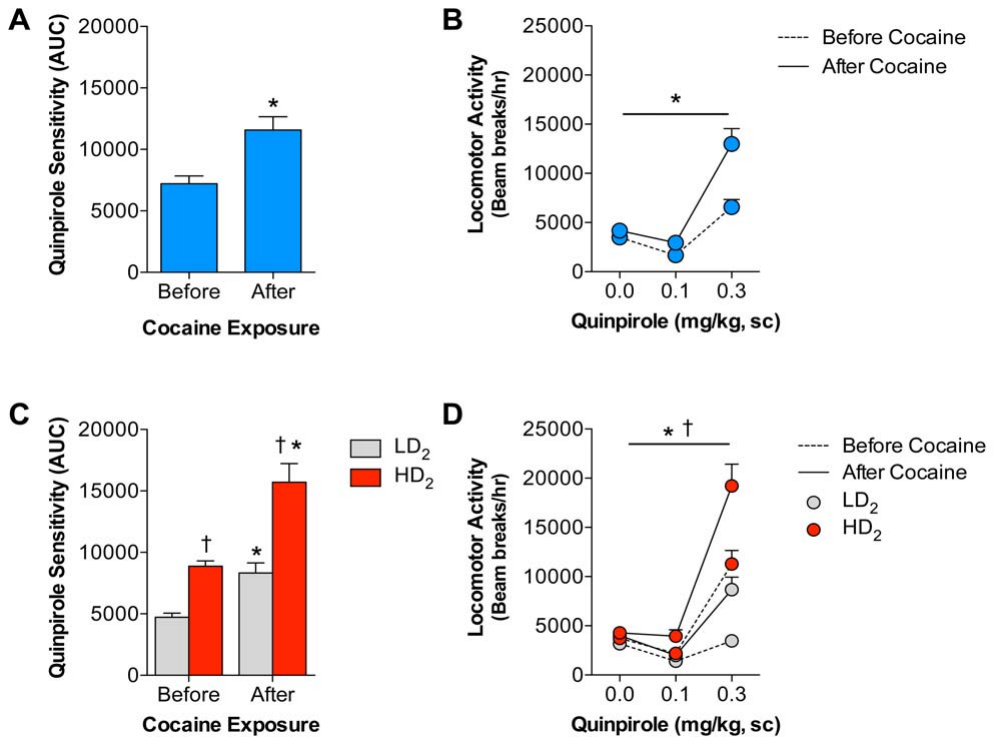
Cocaine self-administration enhances D_2_ DA receptor sensitivity in both LD_2_ and HD_2_ rats. (A) Quinpirole AUC scores were enhanced across the entire cohort of animals tested following cocaine self-administration. *After cocaine significant from Before cocaine, p<0.05 (B) Likewise, this enhancement was observed across all quinpirole doses. *After cocaine significant from Before cocaine, p<0.05. (C and D) Cocaine-induced enhancements in D_2_ DA receptor sensitivity were apparent in both the LD_2_ and HD_2_ groups using both the quinpirole AUC scores and raw locomotor scores across the quinpirole dose response curve. *After cocaine significant from Before cocaine, p<0.05. Interestingly, the group differences persisted even after cocaine exposure. † HD_2_ significant from LD_2_, p<0.05.

It is well established that chronic cocaine treatments increase the sensitivity of D_2_ DA receptors [12], [13], [14], [15]. Therefore, we tested quinpirole sensitivity in all animals following the cocaine self-administration procedure to identify whether the pre-existing differences in D_2_ DA receptor sensitivity persisted following chronic cocaine administration. This was performed in all but 3 animals that were lost due to catheter failure. Figure 7 illustrates that cocaine self-administration enhances quinpirole-induced locomotion compared with responding in the same animals prior to cocaine self-administration. A two-way mixed ANOVA reveals that there was a main effect of cocaine exposure (F_1,104_ = 17.46, p<0.0001) and quinpirole dose (F_2,104_ = 66.73, p<0.0001). There was also a significant interaction (F_2,104_ = 10.61, p<0.0001). Similar results were obtained using the quinpirole AUC scores generated before and after cocaine exposure (t_24_ = 5.56, p<0.0001). We also analyzed the differences between HD_2_ and LD_2_ groups on quinpirole sensitivity before and after cocaine self-administration (Figure 7). Interestingly, pre-existing group differences remained despite cocaine-induced enhancements in D_2_ receptor sensitivity in both groups. Thus, analyses reveal a main effect of group (F_3,98_ = 24.21, p<0.0001), quinpirole dose (F_2,98_ = 117.50, p<0.0001) and the interaction (F_6,98_ = 16.03, p<0.0001). Similarly, results were also obtained using the quinpirole AUC scores generated before and after cocaine exposure. Analyses reveal a main effect of group (F_1,23_ = 46.05, p<0.0001) and cocaine exposure (F_1,23_ = 36.26, p<0.0001), but not the interaction (F_1,23_ = 3.45, p = 0.0760). These findings suggest that even though quinpirole sensitivity prior to cocaine self-administration predicts future cocaine responding, both populations develop quinpirole cross-sensitization following cocaine self-administration.

## Discussion

The findings reported here demonstrate that individual differences in the locomotor responsiveness to quinpirole are predictive of cocaine-induced behavioral regulation. This is the first demonstration that differences in the sensitivity of D_2_ DA receptors predict differential cocaine-induced locomotion, place preference and self-administration. The rats categorized as HD_2,_ having high locomotor activation in response to quinpirole treatments, demonstrate increased cocaine-induced locomotor activity, increased cocaine reward, and self-administer cocaine in greater amounts compared to rats categorized as LD_2_ that have diminished locomotor activation in response to quinpirole. Importantly, categorizations of HD_2_ and LD_2_ did not parallel differences in the exploration of a novel environment, which has been show to be predictive of cocaine responding. Categorizing rats based on their initial cocaine sensitivity (HCR and LCR) did correspond with differences in quinpirole sensitivity suggesting that there may be common mechanisms underlying the individual differences between these two behavioral characteristics. It was determined that the categorization of HD_2_ and LD_2_ did not correspond with the quinpirole-induced suppression of locomotion that is presumably mediated by presynaptic D_2_ DA receptor stimulation [23], [24], [25]. Therefore, we suspect that the HD_2_ and LD_2_ group characterization in quinpirole locomotion likely reflects differences in the sensitivity of postsynaptic D_2_ DA receptors. However, quinpirole is also known to interact with some selectivity at D_3_ DA receptors [32]. In fact, it has been postulated that low doses of quinpirole induce increased oral behavior and yawning behavior in male rats through its interaction with D_3_ DA receptors [33], [34]. Thus, while we speculate that quinpirole-induced locomotion is reflective of postsynaptic D_2_ DA receptor stimulation, it is possible that D_3_ DA receptors may play a role in the behavioral responsiveness to quinpirole.

Alterations within the mesocorticolimbic DA circuitry have been long implicated as both a predisposing factor to psychostimulant use and a consequence of repeated psychostimulant use. The D_2_ DA receptor has received an extraordinary amount of attention due to observations that chronic administration of many drugs of abuse reduces D_2_ DA receptor binding in the striatum, suggesting that drug use produces these changes [6]. However, other lines of evidence suggest that D_2_ DA receptor expression may also correspond to a vulnerability factor. Thus, non-addicted individuals that reported higher drug “liking” scores for methylphenidate also had lower levels of D_2_ DA receptors within the striatum [7]. Using an animal model, it was observed that over-expressing the D_2_ DA receptor in the ventral striatum decreases cocaine self-administration [9]. These findings suggest that expression of D_2_ DA receptors may predict future cocaine use, although neither study address how the sensitivity of the D_2_ DA receptor may correspond with the responsiveness to psychostimulants.

There are several lines of evidence suggesting that the expression levels of metabotropic receptors can be dissociated from the sensitivity of the receptor to initiate intracellular signaling and influence cellular activity. For example, dissociation was observed in rats following a binge-like administration of cocaine. Thus, decreases in D_2_ DA receptor B_max_ were observed suggesting a decrease in D_2_ DA receptor expression following binge cocaine administration, while concomitant increases in G protein activation were observed in response to D_2_ DA receptor stimulation in these same animals [10]. This corresponds with the notion that cocaine self-administration increases the expression of high affinity D_2_ DA receptors without necessarily influencing the overall expression of D_2_ DA receptors [11]. Our studies suggest that individual differences in the behavioral sensitivity to D_2_ DA receptor stimulation predict the responsiveness to cocaine-induced locomotion, reward and reinforcement. Specifically, animals with higher D_2_ DA receptor behavioral sensitivity, whether it is because of greater expression of high affinity D_2_ DA receptors, enhanced G protein activation or another cellular mechanism, predisposes animals to greater cocaine sensitivity, reward and reinforcement. It remains undetermined whether HD_2_ and LD_2_ rats differ in the expression of D_2_ DA receptors and/or G protein activation.

Investigating individual differences as a predictor of drug sensitivity, reward and development of addictive-like behavioral changes has been a long-standing approach to determine vulnerability factors. One of the most established animals models utilizes the habituation response to a novel environment to classify animals as either low or high responders (LR or HR, respectively; [26]). In this model, HR rats exhibit a greater locomotor response to acute cocaine and more readily self-administer low doses of psychostimulants compared to LR rats [26], [27], [35], [36]. Interestingly, HR and LR rats also display differences in D_2_ DA receptor expression where HR rats have decreased B_max_ of ^3^H-raclopride binding and in D_2_ DA receptor mRNA in the nucleus accumbens [37]. These differences are not reflected in the behavioral sensitivity to D_2_ DA receptor stimulation since we did not observed differences between HR and LR rats in quinpirole-induced locomotion confirming previous results [38]. In contrast, an analogous study where rats were selectively bred for differences in responsiveness to novelty, high novelty responders displayed a greater proportion of high affinity D_2_ receptors [39], [40]. Rats bred for high novelty responsiveness also displayed greater quinpirole sensitivity, increased responsiveness to cocaine-related cues and enhanced behavioral disinhibition, findings that are akin to some of our observations. It is unclear whether the differences between HR and LR rats in D_2_ DA receptor expression reflect pre-synaptic or post-synaptic changes or changes in both populations of D_2_ DA receptors. One study reports that HR rats possess subsensitivity of D_2_ autoreceptors in the ventral tegmental area, however it is unknown whether the sensitivity of post-synaptic D_2_ DA receptors in the striatal terminal regions is different between the HR and LR rats [41]. Given some of the inconsistencies in our observations and previous observations we suspect that our D_2_ DA receptor group characterization likely corresponds with mechanisms distinct from generalized locomotor responses to novelty and exploratory behaviors.

Another, more recently developed animal model of individual differences utilizes the initial locomotor response to cocaine to determine HCR and LCR rats [28]. This model has established that LCR rats display greater development of cocaine sensitization [29], enhanced conditioned place preference to cocaine [30], and have higher progressive ratio breakpoints than HCR rats [31]. These findings suggest that animals with a low initial response to cocaine may be more vulnerable to cocaine addiction. We observed that HD_2_ rats have a greater initial response to cocaine, develop cocaine conditioned place preference more readily, and self-administer more cocaine on fixed ratio schedules compared to LD_2_ rats. In an attempt to relate our findings to those using the HCR/LCR characterization, we re-characterized our animals based on their initial cocaine locomotor response. Using this method, we observed that HCR rats had significantly higher D_2_ DA receptor sensitivity compared to LCR rats. While these findings are somewhat contradictory since we find that higher D_2_ DA receptor sensitivity corresponds with behaviors more reminiscent of LCR rats in previous studies (e.g. higher cocaine locomotion, cocaine CPP, increased cocaine self-administration), they are consistent with findings from the Roman high avoidance rat lines where rats that display greater acute locomotor responsiveness self-administer more cocaine [42], [43].

There may be undetermined neurobiological underpinnings that correspond with this discrepancy or it may be a reflection of several experimental differences. First, we did not precisely replicate the published procedures for HCR/LCR characterization. We used a broader characterization of the initial cocaine response. Thus, we collapsed across 2 cocaine doses (5 and 15 mg/kg) and the testing was performed over two hours. This is substantially different than the 30-minute assessment following 10 mg/kg cocaine that was used in previous HCR/LCR studies. Second, the cocaine locomotor testing was performed after the initial quinpirole sensitivity assessment in the same locomotor activity chambers. It is unclear how this experience may have confounded the subsequent cocaine locomotor testing. Finally, we used different procedures in assessing conditioned place preference (ip vs iv cocaine injections) and our self-administration studies were performed after substantial sucrose self-administration. In fact, another recent study utilizing food training prior to cocaine self-administration observed effects more reminiscent of our findings suggesting that this may be an important procedural consideration [44]. In all, these procedural differences may impair our ability to directly compare our studies with those using the HCR/LCR characterization.

Regardless, enhanced initial sensitivity to D_2_ DA receptor stimulation may reflect a vulnerability factor that contributes to increased psychostimulant use. Our observations exploit differences in D_2_ DA receptor sensitivities in an outbred, drug-naïve population of rats. It is possible that genetic or environmental factors could influence D_2_ DA receptor sensitivity rendering some individuals vulnerable or resistant to the behavioral effects of psychostimulants. For example, rearing conditions and social hierarchies have been shown to influence the expression of D_2_ DA receptors. Isolation housing is associated with decreased D_2_ DA receptor expression [45], although others report no change in receptor expression and no change in the behavioral sensitivity of D_2_ DA receptors [46]. In socially housed animals, social dominance can influence the expression of D_2_ DA receptors where dominant animals display increased D_2_ DA receptor expression and are resistant to cocaine self-administration [47], [48]. Given that our animals were individually housed, social hierarchies were likely not a contributing factor, although early life social and/or stressful experiences may have impacted D_2_ DA receptor sensitivities [49], [50], [51], [52], [53], [54], [55].

In summary, we demonstrate that rats with a high initial sensitivity to the locomotor effects of D_2_ DA receptor stimulation, HD_2_ rats, correspond with greater sensitivity to cocaine locomotor sensitivity, cocaine reward, and cocaine taking compared with LD_2_ rats having low initial sensitivity to the locomotor effects produced by D_2_ DA receptor stimulation. This is the first demonstration that D_2_ DA receptor sensitivity is a phenotype representing higher susceptibility to cocaine use, given the exacerbation of cocaine’s behavioral effects. Future studies will be aimed at identifying whether D_2_ DA receptor sensitivity is associated with greater development of behavioral sensitization and cocaine dependence phenotypes as well as associated alterations within the neurobiology of the mesocorticolimbic DA system.

## Supporting Information

Figure S1 **Distribution of quinpirole-induced locomotion in one cohort of animals.** (A) Distribution of locomotor activity scores (beam breaks/hr) during the ascending within-session quinpirole dose response testing. Dark gray horizontal lines within the data clusters depict the median score at each dose. (B) Distribution of the calculated area under the curve (AUC) score for each animal across the three quinpirole doses. The dark gray filled data point and the dotted line represent the median score (*M* = 15460).
(TIF)

Figure S2 **LD_2_ and HD_2_ groups did not differ in their D_2_ dopamine autoreceptor sensitivity.** (A) Distribution of the calculated scores (% Baseline) for 0.1 mg/kg quinpirole within the LD_2_ and HD_2_ groups. Baseline activity corresponds with saline-induced locomotor activity the hour prior to 0.1 mg/kg quinpirole administration in the within session dose response testing procedure. (B) Group averages (± sem) for the D_2_ autoreceptor sensitivity scores revealed not significant group differences.
(TIF)
