# Supplementary figures and images for: Initial D2 Dopamine Receptor Sensitivity Predicts Cocaine Sensitivity and Reward in Rats
Source: PLoS One. 2013 Nov 4;8(11):e78258. doi: 10.1371/journal.pone.0078258 (PMC3817276; doi:10.1371/journal.pone.0078258)

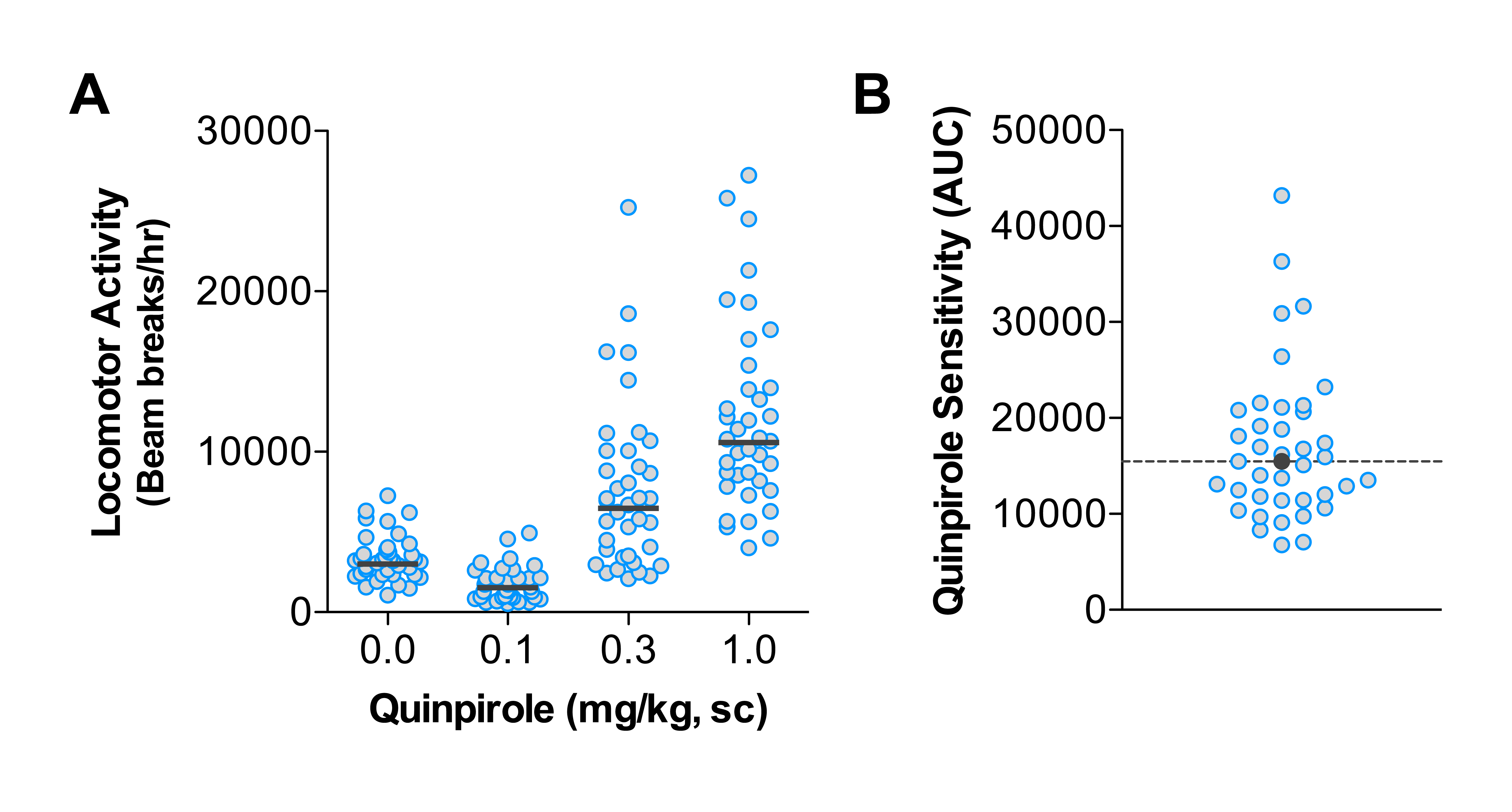

Supplement: Figure S1 — Distribution of quinpirole-induced locomotion in one cohort of animals. (A) Distribution of locomotor activity scores (beam breaks/hr) during the ascending within-session quinpirole dose response testing. Dark gray horizontal lines within the data clusters depict the median score at each dose. (B) Distribution of the calculated area under the curve (AUC) score for each animal across the three quinpirole doses. The dark gray filled data point and the dotted line represent the median score (M = 15460). (TIF) [file pone.0078258.s001.tif]

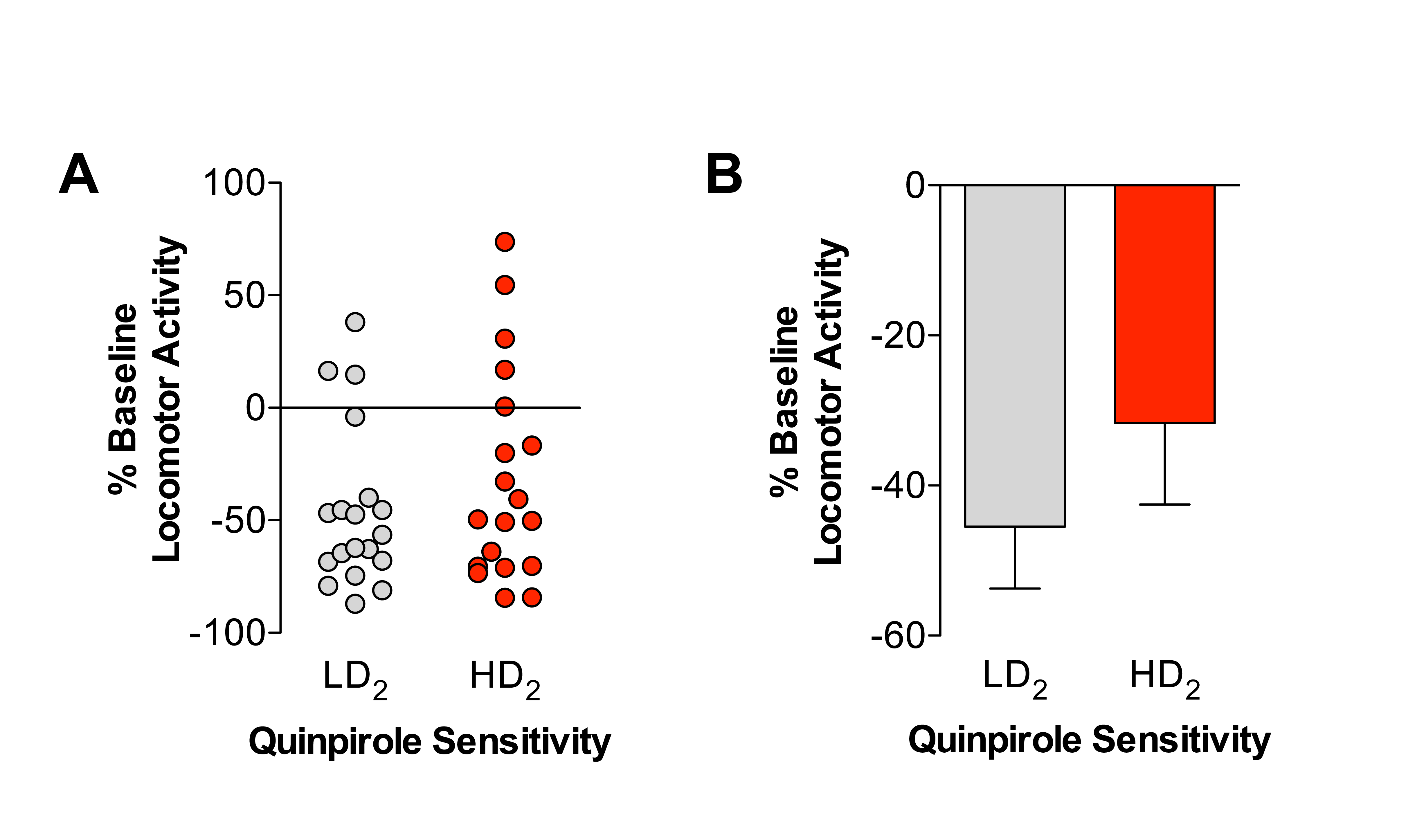

Supplement: Figure S2 — LD2 and HD2 groups did not differ in their D2 dopamine autoreceptor sensitivity. (A) Distribution of the calculated scores (% Baseline) for 0.1 mg/kg quinpirole within the LD2 and HD2 groups. Baseline activity corresponds with saline-induced locomotor activity the hour prior to 0.1 mg/kg quinpirole administration in the within session dose response testing procedure. (B) Group averages (± sem) for the D2 autoreceptor sensitivity scores revealed not significant group differences. (TIF) [file pone.0078258.s002.tif]
